# Hepatokines as a Molecular Transducer of Exercise

**DOI:** 10.3390/jcm10030385

**Published:** 2021-01-20

**Authors:** Dae Yun Seo, Se Hwan Park, Jubert Marquez, Hyo-Bum Kwak, Tae Nyun Kim, Jun Hyun Bae, Jin-Ho Koh, Jin Han

**Affiliations:** 1 National Research Laboratory for Mitochondrial Signaling, Department of Physiology, BK21 Plus Project Team, College of Medicine, Smart Marine Therapeutics Center, Cardiovascular and Metabolic Disease Center, Inje University, Busan 47392, Korea; sdy925@gmail.com (D.Y.S.); jcuevas.marquez@gmail.com (J.M.); kimtn031@gmail.com (T.N.K.); 2 School of Kinesiology, Yeungnam University, Gyeongsan 38541, Korea; psh8179@gmail.com; 3 Department of Kinesiology, Inha University, Incheon 22212, Korea; kwakhb@inha.ac.kr; 4 Institute of Sport Science, Seoul National University, Seoul 08826, Korea; baexx068@snu.ac.kr; 5 Department of Physiology, College of Medicine, Yeungnam University, Daegu 42415, Korea; jinhokoh@yu.ac.kr

**Keywords:** exercise, hepatokines, FGF21, fetuin-A, angiopoietin-like protein 4, follistatin

## Abstract

Exercise has health benefits and prevents a range of chronic diseases caused by physiological and biological changes in the whole body. Generally, the metabolic regulation of skeletal muscle through exercise is known to have a protective effect on the pathogenesis of metabolic syndrome, non-alcoholic fatty liver disease (NAFLD), type 2 diabetes (T2D), and cardiovascular disease (CVD). Besides this, the importance of the liver as an endocrine organ is a hot research topic. Hepatocytes also secrete many hepatokines in response to nutritional conditions and/or physical activity. In particular, certain hepatokines play a major role in the regulation of whole-body metabolic homeostasis. In this review, we summarize the recent research findings on the exercise-mediated regulation of hepatokines, including fibroblast growth factor 21, fetuin-A, angiopoietin-like protein 4, and follistatin. These hepatokines serve as molecular transducers of the metabolic benefits of physical activity in chronic metabolic diseases, including NAFLD, T2D, and CVDs, in various tissues.

## 1. Introduction

Exercise plays a role in regulating whole-body homeostasis, resulting in the improvement and prevention of chronic metabolic diseases, such as metabolic syndrome, hypertension, type 2 diabetes (T2D), and non-alcoholic fatty liver disease (NAFLD) [1]. Exercise-induced skeletal muscle contraction regulates energy metabolism, as skeletal muscles communicate with several key metabolic organs. It is well known that exercise induces the secretion of proteins (myokines) from skeletal muscle, which improve inflammation, mitochondrial function, and insulin sensitivity [2,3,4]. These results indicate that myokines are one of the most important targets for improving lifespan.

Similarly, recent studies have suggested that hepatokines, proteins secreted by the liver, can directly affect chronic metabolic diseases by modulating signaling pathways associated with energy metabolism [5,6]. Several hepatokines have been identified, including fibroblast growth factor 21 (FGF21), fetuin-A, angiopoietin-like protein 4 (ANGPTL4), and follistatin (FST). These hepatokines are key regulators of metabolic organs, including skeletal muscle, the heart, and the brain, resulting in the enhancement of mitochondrial function, and thereby play an inactive role in the development of chronic diseases, such as obesity, cardiovascular disease, T2D, steatohepatitis (NASH), and non-alcoholic fatty liver disease (NAFL) [7,8,9,10,11]. Although these hepatokines function as novel targets for modulating energy metabolism, the mechanism underlying the effect of exercise-induced hepatokines for energy metabolism remains unclear. Therefore, this review aimed to present an update on the role of hepatokines—the molecular transducers of the metabolic benefits of physical activity—in chronic metabolic diseases, including obesity, NAFLD, T2D, and cardiovascular disease (CVD), in various tissues.

## 2. Hepatokines and Disease

The liver is a major organ for maintaining energy metabolism via the regulation of energy storage and utilization under various metabolic conditions, including exercise, fasting, diet, obesity, diabetes, metabolic syndrome, and liver dysfunction [12]. Accumulating evidence shows that hepatokines, which are secreted by the liver, are involved in metabolic-related disease and play a key role in the regulation of NAFLD [13,14]. Xiong et al. [14] evaluated the hepatic transcriptome and proteome in high-fat-diet-induced (HFD) NASH mice. They suggested that the expression of 156 target mRNAs and proteins showed a significant change in the NASH mice. They also provided evidence that the hepatic transcriptome and secretome were completely reprogrammed, resulting in the secretion of hepatokines, which could promote metabolic homeostasis in the HFD-induced NASH mice. In line with this, Yu et al. [15] found that selenoprotein P was upregulated in NAFLD and obese patients, resulting in an inhibition of vascular endothelial growth factor (VEGF)-induced cell proliferation, renal tubule formation, and cell migration. These studies clearly demonstrate not only the central role of hepatokines in the metabolism, but also their role in the occurrence and progression of metabolic diseases, including obesity, insulin resistance, T2D, NAFL, and NASH. To date, hepatokines have been reported to modulate energy metabolism by the direct activation of the liver or peripheral target tissues (Table 1), including skeletal muscles and adipose tissues, and to be associated with inflammation, mitochondrial function, and CVD. The role of the various hepatokines in various disease conditions are discussed below.

## 3. Hepatokines: Molecular Transducers of the Effects of Exercise

Previous studies have demonstrated that the secretion of exercise-regulated factors has a direct effect on the improvement of metabolic disease [12]. Exercise can modulate the accumulation and secretion of adipokines and hepatokines, thereby exerting effects on metabolic diseases. An analysis of the metabolomic changes in mice liver after non-exhaustive exercise revealed the upregulation of 55 genes and a reduction in 29 transcripts after exercise. Among those regulated during acute exercise were FGF21, FST, ANGPTL4, and inhibin E. Interleukin-6 (IL-6), released by exercise-induced skeletal muscles contraction, leads to glucose production in the liver to supply the skeletal muscle contraction. Thus, IL-6, secreted from muscle, is perceived as a sensor of cellular energy status that stimulates the release of energy substrates [13]. Furthermore, aerobic exercise improves leptin levels but does not affect the adiponectin levels in patients with T2D [14]. A study by Tsuzuki et al. [15] reported that aerobic exercise during a ten-week period increased heat shock protein 72 expression in the liver and skeletal muscle, resulting in an improvement in whole-body insulin resistance and lipid metabolism in rats with T2D. Another meta-analysis study demonstrated that acute exercise can transiently increase circulating FGF21 concentration after acute exercise, regardless of weight [27]. FGF21 and FST were also observed to be transiently increased after acute aerobic exercise, wherein FGF21 was increased in an exercise intensity-dependent manner, whilst the increase in FST may be independent of exercise intensity [28].

Additionally, Ko et al. [24] showed that asprosin expression, as a novel hepatokine, is lower in the liver of STZ-injected rats during eight weeks of aerobic exercise. These results suggest that a decreased hepatic asprosin level contributes to the amelioration of diabetes-related parameters through the AMP-activated protein kinase (AMPK)/TGF-β-related pathways and the mitochondria-related pathway through PKA for the potential therapeutic treatment of type 1 diabetes. Another study carried out in a murine model reported that a deficiency of selenoprotein P-upregulated reactive oxygen species and AMPK in skeletal muscle in response to exercise. Taken together, these results suggest that hepatokine production and secretion could be used as a biomarker for monitoring regular exercise-mediated metabolic improvements [6]. The following subsections provide a summary of recent advancements in using hepatokines as molecular transducers of the effects of exercise on metabolic diseases (Table 2).

### 3.1. FGF21

FGF21 is a hepatic protein that serves as a major potential regulator of glucose homeostasis. FGF21 exerts its biological effects through fibroblast growth factor receptor 1c and β-klotho [34]. Jimenez et al. [35] found that patients with T2D and NAFLD presented an increase in their levels of circulating FGF21. Based on these results, they suggested that FGF21 may be an effective novel biomarker for the diagnosis of metabolic disorders. Additionally, the hepatic triglyceride (TG) level has been shown to be the strongest determining factor for FGF21 production and its circulating levels, independent of body mass index. Further, an earlier study reported that a high fructose intake activates carbohydrate-responsive element-binding protein and can upregulate FGF21 production in the liver of humans and mice [36]. Besides this, mice with conditional FGF21 deficiency and FGF21 knock out (KO) showed an impaired glucose metabolism and abnormal body weight, while in FGF21 KO mice a ketogenic diet resulted in severe hepatic insulin resistance compared to that in the wild-type (WT) control. This was associated with increased hepatic diacylglycerol levels and the activation of protein kinase C ε, which is involved in impaired insulin signaling. FGF21 KO mice also presented with an increased incidence of fatty liver disease and very-low-density lipoprotein receptor levels via the activation of the eIF2a-activating transcription factor 4 (ATF4) pathway [37,38,39].

In recent years, numerous observational studies have reported that FGF21 plays a role in metabolic homeostasis [40]. For example, Berglund et al. [41] showed that 3–7 days of subcutaneous FGF21 administration significantly reduced the levels of blood glucose, triglycerides, and fasting insulin, improving glucose metabolism in diabetes animal models. In accordance with these findings, 12 weeks of FGF21 administration reduced body weight and TG levels and improved glucose tolerance, while two weeks of FGF21 treatment significantly reduced liver dysfunction in monkeys with HFD-induced obesity [42]. Further, FGF21 treatment inhibited lipid synthesis in HFD-induced mice by reducing the levels of sterol regulatory element-binding transcription factor-1 and fatty acid synthase. Moreover, 8 days of FGF21 treatment improved hepatic insulin sensitivity in a leptin-deficient obese mouse model [43]. In a NASH mouse model, 3 weeks of FGF21 and FGF21 analog LY2405319 treatment reduced oxidative stress and liver weight [44,45]. In a clinical trial, LY2405319 administration reduced low-density lipoprotein, cholesterol, and TG levels and improved fasting insulin levels in obese human subjects with T2D [46].

### 3.2. The Effects of Exercise on FGF21

A previous study by He et al. [47] demonstrated that exercise had a direct effect on FGF21 expression, evidence that FGF21 is an exercise-mediated myokine. Exercise increased systemic FGF21 levels in transgenic mice overexpressing Akt [48]. However, recent studies have questioned these previous results. For instance, Hansen et al. [49] analyzed the level of FGF21 in the liver and femoral vein of healthy males during chronic exercise and in the recovery phase. They detected hepatic-secreted FGF21 during exercise and in the recovery phase but did not detect FGF21 in the femoral vein. Interestingly, aerobic exercise increased the FGF21 mRNA expression in the liver, but not in skeletal muscles [49,50]. Further, following the injection of glucagon and somatostatin into healthy males, a significant increase in splanchnic FGF21 levels was observed compared to levels in the control group [49]. Similarly, exercise suppressed the FGF21 mRNA expression in the liver of glucagon receptor-KO mice [51]; moreover, exercise induced an increase in the plasma insulin levels but did not affect the release of FGF21 into circulation. In healthy males, circulating FGF21 has been shown to regulate the levels of FFA during exercise [32]. Moreover, in palmitic acid-treated FaO cells, FGF21 transcription was found to be upregulated by ATF4/peroxisome proliferator-activated receptor alpha (PPARα), suggesting the involvement of the ATF4/PPARα pathway in the promotion of hepatic FGF21 production during exercise-mediated lipid degradation [52]. However, recent reports have shown that the increased plasma FGF21 during exercise is suppressed in patients with T2D [53]. Therefore, it can be summarized that exercise-mediated FGF21 production is different in metabolic diseases.

Previous studies have evaluated the effects of chronic exercise on FGF21 levels in patients with metabolic diseases. Endurance exercise with dietary interventions has been reported to significantly reduce the levels of circulating FGF21 in elderly patients with obesity [54]. However, this result was inconsistent with that of another study on patients with obesity and diabetes [55]. These conflicting results may be explained by methodological issues. Firstly, concerning metabolic diseases, these studies were conducted on heterogeneous groups. Secondly, factors affecting the systemic FGF21 levels, including nutritional intake, fasting, and circadian rhythms, were not considered. Finally, none of the previous studies determined changes in insulin, FFAs, and hepatic fat levels, nor evaluated cardiovascular function, which plays a role in the regulation of FGF21 levels. Fletcher et al. [56] examined the effects of a voluntary wheel-running exercise on FGF21 in a T2D model using Otsuka Long-Evans Tokushima Fatty (OLETF) rats, a spontaneously diabetic rat model. Their results showed that exercise-induced OLETF rats maintained their hepatic FGF21 mRNA and circulating FGF21 levels compared to mice in the control group. However, in FGF21-KO mice a voluntary wheel-running exercise reduced fat, inflammation, hyperinsulinemia, and the hepatic fatty acid level [57]. Contrary to these findings, in HFD mice the same exercise protocols did not show any effect on glucose tolerance or hepatic TG levels [50], but had positive effects on body weight and inhibited increases in fat mass independent of genotype [58]. This led to the conclusion that FGF21 KO caused a diminished response to exercise and reduced AMPK activation in skeletal muscles.

Taken together, these studies suggest that FGF21 may play a role in glucose and lipid metabolism, resulting in the improvement of the metabolic diseases. Although the beneficial effects of exercise on chronic diseases are well known, the underlying mechanisms are unclear; thus, exercise-induced hepatic FGF21 production may activate exercise-mediated effects in various tissues, suggesting that FGF21 promotes inter-organ crosstalk in metabolic diseases.

### 3.3. Fetuin-A

Fetuin-A, a hepatokine and an α2-Heremans-Schmid glycoprotein (AHSG), is secreted from both the liver and adipose tissues [59]. Fetuin-A was initially identified as a physiological inhibitor in the liver and skeletal muscle [60]. Recently, many studies have demonstrated the role of fetuin-A in metabolic diseases. For example, Chung et al. [61] suggested that fetuin-A may also serve as a marker for metabolic disease. Wang et al. [21] observed that high levels of plasma fetuin-A were strongly associated with T2D in the Chinese population. Moreover, fetuin-A plays an important role in glucose tolerance, insulin resistance, liver fibrosis, and T2D; however, a direct correlation with hepatic fat accumulation has yet to be demonstrated [62,63]. Fetuin-A has been shown to induce metabolic dysfunction in transgenic mice, while its KO resulted in improved glucose tolerance and insulin sensitivity in response to HFD. These results suggested that fetuin A modulates Akt and mitogen-activated protein kinase, resulting in an activation of the phosphorylation of the insulin receptor and downstream signaling [64]. Similarly, a single dose of fetuin-A injection was shown to inhibit both insulin receptor and insulin receptor substrate-1 phosphorylation, resulting in the development of insulin resistance [65,66]. In fact, insulin resistance animal models have consistently demonstrated that fetuin-A is overexpressed in metabolic disorders, and HFD-induced models were shown to exhibit fetuin-A mRNA expression in vivo [66,67]. Clinically, the expression of the E3 ubiquitin-protein ligase, FBXW7, which plays a role in the ubiquitination and degradation of fetuin-A, is significantly reduced in the liver of obese patients [68]. Takata et al. [69] also demonstrated that the fetuin-A level increased following glucose infusion and that *AHSG* transcription was upregulated in HepG2 cells via the ERK1/2 signaling pathway. Similarly, an increase in fetuin-A expression and secretion in HepG2 cells was observed following incubation with palmitate via NF-κB activation [70]. Fetuin-A also acted as an endogenous ligand for Toll-like receptor 4 (TLR4), which contributes to the free fatty acid (FFA)-induced insulin resistance in adipocytes, macrophage invasion, and inflammation. Likewise, the activation of TLR4 induces insulin resistance in adipocytes [71]. TLR4 KO mice are protected from lipid treatment- or HFD-induced insulin resistance. Moreover, fetuin-A has been shown to stimulate lipotoxicity in β cells via TLR4 signaling, which interferes with glucose-stimulated insulin secretion. Finally, fetuin-A promotes insulin resistance, thereby inhibiting the activation of insulin receptor tyrosine kinase [72]. In summary, these results suggest that fetuin-A can modulate the insulin signaling pathways, contributing to metabolic diseases.

### 3.4. The Effects of Exercise on Fetuin-A

Although little information exists on the effects of exercise (acute or chronic) on fetuin-A expression, there has been increased interest in the exercise physiology field regarding future transducers of the effects of exercise on metabolic diseases. A recent study by Sargeant et al. [73] showed that 60 min of aerobic exercise (cycling/treadmill) did not have any effect on the systemic levels of fetuin-A in obese and healthy adults. In another study, patients with obesity showed a rapid increase in serum phospho-fetuin-A (Ser312) after a session of exercise, which returned to baseline levels within 24 h. Within 24 h of exercise, the glucose and insulin levels decreased, suggesting that a single session may modulate fetuin-A levels [74]. Similarly, Sakr et al. [75] reported that 16 weeks of swimming exercise decreased serum fetuin-A levels and improved the homeostatic model assessment for insulin resistance (HOMA-IR) scores in a metabolic syndrome model. Malin et al. [76] also reported that endurance exercise could decrease fetuin-A levels in obese patients with NAFLD. This result suggested that exercise improved glucose tolerance and insulin resistance. Similar to the observational findings, 12 weeks of aerobic exercise reduced the plasma fetuin-A level [77]. Interestingly, the effects of exercise in reducing fetuin-A levels were not accompanied by changes in the hepatic TG levels, suggesting that exercise-induced fetuin-A could be related to changes in blood lipids rather than to hepatic lipids [78]. In line with this, Lee et al. [79] reported that 12 weeks of exercise decreased the fetuin-A level and improved glucose metabolism in patients with dysglycemia. They also reported that decreased plasma fetuin-A levels can predict alterations in the expression of genes associated with inflammatory TLR signaling in macrophages and adipose tissue. Based on this evidence, exercise-induced decreases in fetuin-A via the downregulation of TLR4 may serve as the underlying mechanism of the observed exercise-induced anti-inflammation in patients with obesity, NAFLD, NASH, and T2D. Moreover, since regular exercise can reduce the levels of circulating fetuin-A, it may improve insulin sensitivity in patients with metabolic disorders.

### 3.5. ANGPTL4

ANGPTL4 is a secreted protein belonging to the angiopoietin-like protein gene family and is highly expressed in adipose tissues and the liver in both humans and mice; it is also, to a lesser extent, expressed in other tissues, such as the heart and muscles. The expression levels of ANGPTL4 in these tissues or organs affect metabolic regulation during fasting and hypoxia [80,81]. Recent studies have indicated that plasma ANGPTL4 also regulates the clinical relevance of metabolic diseases and is positively correlated with the plasma levels of FFA [81]. ANGPTL4 has been shown to regulate lipid metabolism via stimulating lipid degradation and inhibiting lipoprotein lipase (LPL) activity in adipocytes [78], as LPL is responsible for hydrolyzing the TG core of circulating TG-rich lipoproteins and is capable of storing or oxidizing FFA [82]. The overexpression of ANGPTL4 in mice is associated with reduced LPL activity as well as increased circulating TG and cholesterol levels compared to control animals [83]. For instance, adenovirus-mediated ANGPTL4 overexpression has been shown to improve glucose tolerance and reduce hepatic glucose output [84]. In another study, Lichtenstein et al. [85] suggested that the overexpression of ANGPTL4 in transgenic mice caused impaired glucose utilization and reduced insulin-mediated hepatic glucose metabolism. In fact, Janssen et al. [86] reported that ANGPTL4-KO mice showed improved glucose tolerance, although increased fat mass, visceral fat, and inflammation were observed. Moreover, ANGPTL4 deficiency in adipose tissues leads to an improved glucose metabolism and lowered lipid accumulation in the liver and skeletal muscle. Taken together, ANGPTL4 improved adipose tissues and lipid metabolism to exert beneficial effects on the lipid and glucose metabolism.

### 3.6. The Effects of Exercise on ANGPTL4

The first study to report the effects of exercise intervention on ANGPTL4 was performed by Kersten et al. [87]. They found that an aerobic exercise intervention (2 h/cycling/50% VO_2_ max) increases the circulating levels of ANGPTL4 during fasting in healthy adults. Moreover, Ingerslev et al. [5] reported an increase in ANGPTL4 mRNA expression after a single session of one-legged exercise (50% of the maximum workload, 60 min). These studies elucidated the mechanisms underlying exercise-induced ANGPTL4 secretion by assessing arterial-to-venous differences in the leg and the hepato-splanchnic bed. The authors suggested that the exercise-induced increase in systemic ANGPTL4 levels should be attributed to the liver rather than to the contracting muscles. Further, employing the pancreatic clamp technique to suppress increases in the glucagon/insulin ratio and FFAs suppressed the secretion of ANGPTL4. These results suggest that the glucagon/insulin ratio and FFAs are pivotal in ANGPTL4 secretion. Moreover, in hepatocytes the glucagon/insulin ratio, modulated by the activation of the cAMP-PKA pathway, has been shown to upregulate ANGPTL4 transcription. Hence, it can be summarized that although ANGPTL4 is an exercise-induced hepatokine, its increased plasma levels do not originate from skeletal muscles. Nevertheless, ANGPTL4 secretion from skeletal muscles during exercise may still act in an autocrine manner [31]. Although a single session of aerobic exercise has been shown to upregulate ANGPTL4 mRNA expression in mouse liver, it remains unclear whether hepatocytes can contribute to its increased levels in plasma.

There is little information on whether endurance exercise affects systemic levels of ANGPTL4. Recent studies on ANGPTL4 have emphasized the need to consider its role in metabolic diseases. For example, Catoire et al. [31] reported that 12 weeks of endurance exercise elicited no significant effects on the systemic levels of ANGPTL4 in healthy adults. However, another report showed that, in obese individuals, 6 months of endurance exercise decreased weight and increased the levels of systemic ANGPTL4 [88]. It is well known that physical activity leads to short- and long-term adaptation to the energy demand of the body, which is supported through lipid metabolism. Although ANGPTL4 modulates LPL activity and is important in fine-tuning lipid metabolism in response to physical activity, further studies are required to verify whether exercise-induced ANGPTL4 secretion mediates the benefits of exercise for metabolic diseases. Taken together, exercise-induced ANGPTL4 plays a role in regulating lipid metabolism in metabolic diseases.

### 3.7. FST

FST is a glycosylated plasma protein belonging to the transforming growth factor beta (TGF-β) superfamily. FST is expressed in the liver, skeletal muscles, and white and brown adipose tissue [89,90]. The basal expression levels of FST have been shown to increase in patients with T2D, NAFLD, and NASH. Further, increased FST levels have been found to positively correlate with HbA1c and impaired fasting blood glucose levels [91,92]. For example, Polyzos et al. [92] found that blood FST levels may contribute to the progression of NAFLD to NASH in patients with obesity, NAFLD, and NASH, as well as in healthy individuals. Moreover, a recent study reported that bariatric surgery in obese diabetic patients significantly reduced the FST levels and improved the HbA1c levels [93]. Similarly, in vivo and in vitro studies have confirmed a central role of FST, which is regulated by the glucagon-to-insulin ratio during exercise dependent on cAMP in hepatocytes [94,95]. FST is also reported to be involved in the regulation of systemic metabolic abnormalities caused by FoxO1 activation. In addition, the adenovirus-mediated overexpression of FST315 in HFD mice impaired blood glucose response in an oral glucose-tolerance test [96]. However, another study reported improved steatosis through the activation of hepatic insulin signaling pathways in FST315-KO mice [97]. Tao et al. [78] also demonstrated that the suppression of FST led to improved glucose tolerance and insulin levels, thereby confirming the crucial role of FST in hepatic glucose metabolism. Moreover, in white adipose tissues under hyperinsulinemic clamp, insulin sensitivity improved via increased Akt signaling, while hepatic glucose output decreased. Therefore, although FST may be induced in the development of metabolic diseases, further clinical research is required to elucidate its precise role in metabolic homeostasis.

### 3.8. The Effects of Exercise on FST

Previous studies have shown that a single exercise session induces FST release into the bloodstream [98]. Specifically, Hansen et al. [99] revealed that long-term moderate-intensity cycling increased the blood FST levels in healthy adults but did not modulate the FST mRNA expression in the vastus lateralis. Similarly, swimming exercise significantly increased the FST protein and mRNA levels in adipose tissues, but not in skeletal muscles [94]. Moreover, although somatostatin-glucagon combination treatment increased the plasma FST levels, exercise-induced FST secretion was partially blunted during the pancreatic clamp. This supports the finding that while glucagon increases FST production in hepatocytes, insulin suppresses it via cAMP. Although the acute exercise-mediated regulation of FST has only been partially characterized, a dearth of scientific data exists on the effects of chronic exercise on FST [99]. Among related studies, resistance exercise increased blood FST levels in overweight elderly women, while high-intensity interval exercise led to higher FST levels in the sedentary elderly [94,99].

Recurrent physical activity has been established to regulate glucose metabolism and insulin sensitivity. Although exercise-induced FST is a hepatokine, much is not known about its physiological role in response to long-term regular exercise. Nevertheless, based on the available scientific literature, it can be surmised that FST may contribute to cellular adaptation to exercise and may assist in the prevention of metabolic diseases. Moreover, as FST contributes to the development of muscle hypertrophy, it may also be capable of promoting exercise-induced inter-organ crosstalk.

## 4. Crosstalk Between Hepatokines and Lipogenesis

The effects of exercise or physical activity prevented metabolic disorders and lipogenesis. FGF-21, Fetuin-A, ANGPTL4, and FST were observed to be involved in the specific mechanisms between hepatokines and lipogenesis [100]. For example, whole-body FGF21-KO mice had impaired glucose metabolism and an excessively abnormal body weight [37]. The FGF21-KO mice increased hepatic steatosis and low levels of lipoprotein receptor protein via the activation of the eIF2a-ATF4 pathway [39]. The beneficial effect of FGF-21 decreased low-density lipoprotein cholesterol and TG, increased high levels of lipoprotein cholesterol, and improved fasting insulin [46]. Fetuin-A plays an important role in the regulation of lipogenesis. Fetuin-A-KO mice had an improved glucose clearance rate and were protected against HFD-induced metabolic disorder. These mechanisms were associated with a higher insulin-stimulated phosphorylation of insulin receptor and down-regulated MAPK and Akt in the liver [64]. In addition, Steiger et al. [101]. provided evidence that the level of plasma ANGPTL4 was associated with obesity in humans. In an animal model, the overexpression of ANGPTL4 in mice increased their triglycerides, cholesterol, and LPL activity [83]. The ANGPTL4 also affected glucose homeostasis and metabolic function in obesity-induced mice [86,102]. Tao et al. [103] reported that FST plays a role in the improving of HbA1c in obese patients with diabetes. FST-transgenic mice showed a decrease in abdominal fat, increased glucose clearance, and improved plasma lipid profiles compared to WT mice. These results provided evidence that FST upregulates metabolic biomarkers and lipidomic profiles [104].

Several recent studies have demonstrated that hepatokines contribute to adipogenic homeostasis [105]. The FST, which was highly expressed in brown adipose tissues, improved glucose tolerance and insulin synthesis [105]. Overall, hepatokines including FGF-21, Fetuin-A, ANGPTL4, and follistatin can ameliorate metabolic disorders and lipogenesis in obesity and T2D [106,107]. Therefore, hepatokines are closely linked to lipogenesis and improved metabolic-related factors.

## 5. Future Directions

The effects observed in both clinical and pre-clinical data in response to exercise appear to also be in part of the modulatory role in mitochondrial function, as exercise is known to modify mitochondrial function during homeostasis. The previous sections have discussed how these different hepatokines are responsible for glucose and lipid regulation, in which both processes have mitochondria central to their pathways. Thus, it is also essential that a link between exercise-mediated mitochondrial regulation in relation to hepatokines be established.

FGF21, as a potential stimulus to improve metabolic diseases, is a powerful metabolic regulator of mitochondrial dysfunction. For example, FGF21-KO showed lower levels of hepatic mitochondrial complete palmitate oxidation, β-hydroxyacyl-CoA dehydrogenase (β-HAD) activity, and PGC-1α nuclear content than WT mice during 8 weeks of voluntary wheel-running exercise. The exercise-induced increase in the mRNA expression of *PEPCK*, a hepatic gluconeogenic gene, was found to be lower in FGF21-KO mice than in WT mice [108]. From our review results, FGF21 was upregulated by the ATF4/PPARα signal pathway in the liver during exercise. Plasma FGF21 can be suppressed in patients with T2D. Recurrent physical activity is imperative for the regulation of glucose metabolism and insulin sensitivity. It is well known that ANGPTL4 is highly expressed in skeletal muscle following exercise. For example, ANGPTL4 treatment upregulated the AMPK signaling and improved the mitochondrial respiratory capacity in skeletal muscle. This can explain how ANGPTL4 may be capable of increasing exercise tolerance time via the AMPK-mediated signaling pathway [109]. However, to the best our knowledge, previous studies are lacking in terms of identifying a marker of mitochondrial function. Although fetuin-A, ANGPTL4, and FST have shown positive effects on exercise-related performance, most studies have highlighted the role of physiological variables in skeletal muscle. Therefore, it is necessary to further explore the functions of exercise-induced hepatokines including andropin, FGF21, fetuin-A, ANGPTL4, and FST.

While the aforementioned studies appear promising in terms of the potential of hepatokines in regulating metabolic diseases, several issues remain that must be considered before proceeding with future studies. Related factors such as cytokines and metabolites capable of regulating hepatokine action in target tissues should be considered when examining inter-tissue crosstalk involving extracellular hepatic tissues and tissues that are highly affected during exercises, such as adipose tissues and skeletal muscles. Moreover, circulating hepatokines target different associated factors that elicit positive or negative responses depending on the pathway mechanisms triggered by specific exercises. Thus, more in-depth studies examining the signaling pathways should be conducted [107]. It is also necessary to establish at which concentration or fold increase these hepatokines exert the most beneficial or detrimental effects both physiologically and pathologically. Further, a variety of exercise modalities and conditions exist that can trigger the release of specific hepatokines, the level of which depends on the intensity and duration of exercise to which the subject was exposed. Determining an optimal modality should be further assessed to assist clinicians with the effective prescription of appropriate types of exercise therapy for patients. The studies discussed herein were also provided with various aspects of models and, in some cases, in different stages of pathology. Therefore, an in-depth analysis of these hepatokines during exercise is necessary to unveil their benefits in ameliorating disease progression and the onset of disease [100].

## 6. Conclusions

**Figure 1:**
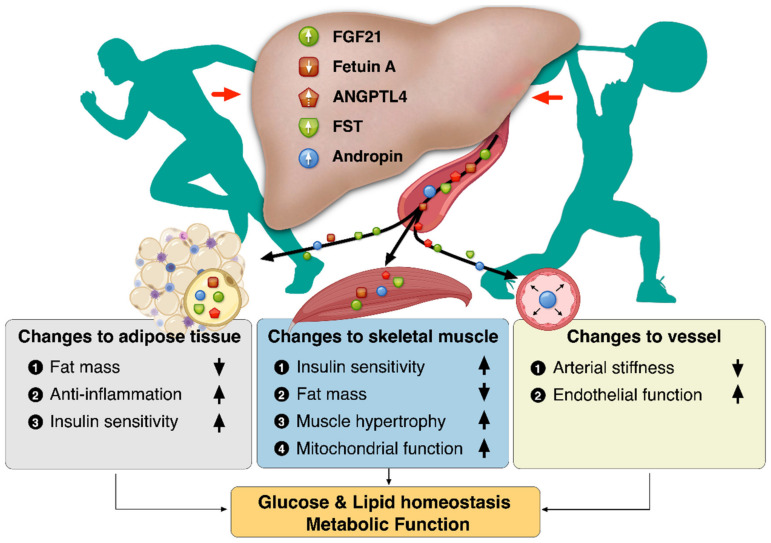
Benefits of exercise-induced hepatokines: changes to the adipose tissue, vessel, and skeletal muscle.

Regular exercise offers protection against chronic metabolic diseases such as NAFLD, T2D, and CVDs. Besides skeletal muscle, several tissues respond to exercise, bringing into play the issue of exercise-induced signaling molecules that could mediate this cross-talk. The liver is not merely an organ receiving humoral stimuli, but also an organ communicating with extrahepatic tissues such as adipose tissue and skeletal muscle through hepatokines. Exercise-induced hepatokines plays a role in regulating energy balance by improving insulin sensitivity, inflammation, and mitochondrial function, thereby contributing to the improvement of metabolic disorders. Exercise-induced hepatokines might also create a paradigm shift in strategies to diagnose and treat chronic metabolic diseases. Collectively, the benefits of exercise-induced hepatokines have revealed changes to the adipose tissue, vessel, and skeletal muscle for metabolic function, as depicted in Figure 1.

## Figures and Tables

**Table 1:** The role of hepatokines in tissues and the blood.

Hepatokines	Subjects	Places	Main Effects	Refs
Tsukushi	Mice	NAFLD	Reduce circulating high-density lipoprotein cholesterol. Lower cholesterol efflux capacity. Decrease cholesterol-to–bile acid conversion in the liver.	Mouchiroud et al. [16]
ANGPTL8	Mice	Liver	Treat metabolic disorders.	Chen et al. [17]
Selenoprotein P	Mice	Skeletal muscle	Inhibit the development of hyperglycemia in type 2 diabetes. Improve insulin resistance.	Misu [18]
LECT2	Mice	Adipocytes	Stimulates inflammation and insulin resistance.	Jung et al. [19]
FGF21	Human	Plasma	Biomarkers of metabolic disease in human.	Keuper et al. [20]
Fetuin-A	Human	Plasma	Positive association between plasma fetuin-A levels and risk of developing type 2 diabetes.	Wang et al. [21]
ANGPTL4	Human	Plasma	Significantly predict cardiovascular events independent of conventional cardiovascular risk factors.	Muendlein et al. [22]
FST	Rat	Skeletal muscle	Increase anabolic, neurotrophic, and the progression of satellite cells.	Isaacs et al. [23]
Asprosin	Rat	Adipose tissue	Protect against hyperinsulinism associated with metabolic syndrome.	Romere et al. [24,25]
Heat shock protein 72	Human	Skeletal muscle	Increase maximal voluntary eccentric contractions of biceps.	Yamada et al. [26]

Abbreviations: ANGPTL8, angiopoietin-like protein 8; LECT2, Leukocyte cell-derived chemotaxin-2; ANGPTL4, angiopoietin-like protein 4; FST, follistatin; NAFLD, non-alcoholic fatty liver disease.

**Table 2:** Effects of exercise interventions on hepatokines.

Hepatokines	Exercise Intervention		Effects	Refs
	Exercise Type	Frequency		
Andropin	Aerobic	90 min, 3-5 days/weeks	Increased serum andropin, thus reducing arterial stiffness and improving endothelial function.	Fujie et al. [29] Zhang et al. [30]
ANGPTL4	Aerobic	120 min, single bout	Selective induction of ANGPTL4 reduces the local fatty acid uptake, while directing fattiy acid to active skeletal muscle as a fuel.	Catoire et al. [31]
Fetuin A, Fetuin B, FGF21	Aerobic	30–45 min/day, 3 days a week, 8 weeks	Prevention of type 2 diabetes. Greater improvement in hepatokines was observed in resistance exercise.	Keihanian et al. [32]
	Resistance	Leg press, bench press, knee extension, seated cable row, knee flexion, military press, and calf rise, 3 days/week, 8 weeks		
FST	Aerobic	12 m/min, 30 min, and increasing up to 30 m/min, 60 min	Both exercises increased FST in rats, which can be of use in addressing muscular disorders.	Rashidlamir et al. [33]
	Resistance	3 sets of 5 repetitions, 3 sessions per week for 8 weeks		

Abbreviations: ANGPTL4, angiopoietin-like protein 4; FGF21, fibroblast growth factor 21; FST, follistatin.
